# The Role of Intra-Amygdaloid Neurotensin and Dopamine Interaction in Spatial Learning and Memory

**DOI:** 10.3390/biomedicines10123138

**Published:** 2022-12-05

**Authors:** Bettina Réka László, Erika Kertes, Tamás Ollmann, László Péczely, Anita Kovács, Zoltán Karádi, László Lénárd, Kristóf László

**Affiliations:** 1 Institute of Physiology, Medical School, University of Pécs, 7602 Pécs, Hungary; 2 Neuroscience Center, University of Pécs, 7602 Pécs, Hungary; 3 Learning in Biological and Artificial Systems Research Group, Institute of Physiology, Medical School, University of Pécs, 7602 Pécs, Hungary; 4 Neuropeptides, Cognition, Animal Models of Neuropsychiatric Disorders Research Group, Institute of Physiology, Medical School, University of Pécs, 7602 Pécs, Hungary; 5 Szentágothai Research Center, Cellular Bioimpedance Research Group, 7624 Pécs, Hungary; 6 Szentágothai Center, Molecular Endocrinology and Neurophysiology Research Group, University of Pécs, 7624 Pécs, Hungary

**Keywords:** amygdala, neurotensin, dopamine, spatial learning, memory, passive avoidance learning

## Abstract

Neurotransmitter and neuromodulator neurotensin (NT) has been proved to facilitate spatial and passive avoidance learning after microinjected into the rat central nucleus of amygdala (CeA). These previous studies of our laboratory also revealed that neurotensin-1 receptor (NTS1) is involved in the mentioned actions of NT. Extensive literature confirms the interaction between neurotensinergic and dopaminergic systems, and our research group also suppose that the mesolimbic dopaminergic system (MLDS) is involved in the spatial learning and memory-facilitating effect of NT in the CeA. In the present work, NT and dopamine (DA) interaction has been examined in the Morris water maze and passive avoidance tests. Rats received 100 ng NT, 5 µg dopamine D2 receptor antagonist sulpiride in itself, sulpiride as a pretreatment before NT or vehicle solution into the CeA. NT microinjection significantly decreased target-finding latency in the Morris water maze test and significantly increased entrance latency in the passive avoidance test, as was expected based on our previous findings. The DA D2 receptor antagonist pretreatment was able to inhibit both effects of NT. The results confirm the facilitatory effect of NT on spatial learning and memory and let us conclude that these actions can be exerted via the DA D2 receptors.

## 1. Introduction

Neurotensin (NT) is a long-known tridecapeptide [1] distributed widely in the central nervous system that acts as a neurotransmitter and neuromodulator and is involved in learning and reinforcement processes [2,3]. From its four types of receptors (NTS1, NTS2, NTS3 and NTS4, respectively [4]), NT was published to have the highest affinity to neurotensin-1 receptor (NTS1) [5,6]. Amygdala, the target brain area of our experiments, known among others for its role in the regulation of learning, memory and fear-related behavior, has been shown to be rich in NT immunoreactive elements and NTS1s [2,7].

The effect of NT on spatial learning has been examined previously by our research group in the Morris water maze test [8]. Bilaterally microinjected, intra-amygdaloid NT significantly reduced escape latency, that is, it facilitated spatial learning in the rat central nucleus of the amygdala (CeA). Pretreatment with NTS1 antagonist SR 48692 was able to block this effect; in this way, the involvement of NTS1 was clarified in this action of NT. Our findings about this role of NT and the involvement of NTS1 were supported by another study [9] where NT or NTS1 agonist PD 149163 microinjected into the rat entorhinal cortex facilitated spatial learning in the Barnes maze test. The effect was similar in wild-type mice, in an Alzheimer’s disease mouse model and in an NTS2 knockout, but not in an NTS1 knockout mouse [9].

In another study of our research group, the role of NT in passive avoidance learning has been examined in the rat CeA [10]. NT caused a significant increase in the entrance latency, and, similarly to the previous experiment, SR 48692 pretreatment blocked the action of NT, that is, NT facilitated passive avoidance learning via NTS1. Post-trial NT microinjection proved to be effective in promoting passive avoidance learning not only in the CeA and the mammillary body, but after its intracerebroventricular application as well [11,12]. Furthermore, NT was shown to be able to prevent impairments in the reproduction of passive avoidance responses caused by reserpine in the nucleus accumbens (NAC) [13].

Considering the growing amount of data confirming that the rewarding and learning facilitating effect of certain neuropeptides, including NT, is based on the modulation of the mesolimbic dopaminergic system (MLDS) [14,15], we assume that same mechanism might be in the background of the spatial learning and memory-facilitating effect of NT in the CeA. The key role of the MLDS in learning, motivation and cognitive processes is well-known [16,17,18]. This pathway system originates from the ventral tegmental area (VTA), which is among the most known action sites of NT in the central nervous system [19,20,21], and innervates among others the amygdala as well [22,23]. It has been revealed that NT receptors of the VTA are mainly located on DAergic neurons [24]. In addition to these findings, a considerable amount of studies have demonstrated the interaction between NTergic and DAergic systems: NT enhances the activity of DAergic neurons in the VTA [25,26] and increases DA release in several brain areas [26,27,28,29,30]. The interaction of intra-amygdaloid NT and DA has already been investigated by our research group in a conditioned place preference test [31]. After verifying the positive reinforcing properties of NT microinjected into the CeA [32], the effects of DA D1 receptor antagonist SCH 23390 and DA D2 receptor antagonist sulpiride were examined [31]. The fact that pretreatment with both the DA D1 and DA D2 antagonist could prevent the positive reinforcing effect of NT supports the assumption that the rewarding effect of NT can be due to the modulation of the DA system.

In the present experiments, the interaction of NT and DA has been studied in the Morris water maze and passive avoidance tests in order to clarify the role of DA in the spatial learning and memory-facilitating effect of NT.

## 2. Materials and Methods

### 2.1. Subjects

A total of 64 adult male Wistar rats weighing 270–290 g were used in the present series of experiments, from which the data of 56 were evaluated. The animals were caged individually and kept on standard laboratory chow pellet food (Charles River Ltd., Budapest, Hungary) and tap water ad libitum in a room with constant temperature (23 ± 2 °C) and humidity (55–60%), as well as with 12–12 h dark/light cycle according to the natural time of the day. Our experiments were performed in accordance with institutional (ethical permission No.: BA 02/2000-72/2017), national (Law XXVIII, 1998, Government Decree, 40/2013. (II.14) Hungary) and international regulations (European Community Council Directive 86/609/EEC; 1986, 2006; European Directive 2010/63/EU of the European Parliament and of the Council; National Institutes of Health Guidelines, 1997). The minimum number of animals needed to evaluate the results was used in our experiments, and all efforts were made to provide the required conditions for their well-being and to minimize their suffering.

### 2.2. Surgery

Guide cannulae were implanted in stereotaxic operation under anesthesia induced by the intraperitoneal (ip.) injection of ketamine supplemented with diazepam (Calypsol and Seduxen, Richter Gedeon, Budapest, Hungary; ketamine: 80 mg/kg body weight, diazepam: 20 mg/kg body weight). The 22 gauge stainless steel guide cannulae were implanted bilaterally, directed toward and 1 mm above the dorsal border of the CeA. Coordinates were determined according to the stereotaxic atlas of the rat brain [33] and were the following: AP: −2.3 mm and ML: ±4.1 mm relative to the Bregma, DV: −6.5 mm. Cannulae were fixed to the skull with three stainless steel screws and dental acrylic. When not being used for injection, guide cannulae were occluded with 27 gauge stainless steel obturators (mandrins). Operations were followed by a minimum 6 days of postoperative recovery period before starting the experiments, during which period animals were handled daily.

### 2.3. Drugs and Injection Procedure

Drugs. NT (Sigma-Aldrich Co. (St. Louis, MO, USA): N 3010, mw.: 1830.72 g/mol) was used in 100 ng dose (54.6 pmol) in the present experiments. NT was dissolved in phosphate-buffered saline (PBS, Veh1): physiological saline solution containing 0.01 M Na-acetate and 0.01 M phosphate buffer. The pH of this solution was 7.4. Control animals received Veh1 in a volume equal to NT microinjections.

Sulpiride (Sigma-Aldrich Co.: S7771(S), mw.: 341.43 g/mol) was used in 5 µg (14.6 nmol) dose as DA D2 antagonist. Sulpiride was dissolved in 0.1 M HCl, and after the addition of phosphate buffer it was titrated with 0.1 M NaOH. The vehiculum produced this way (Veh2) was microinjected to the control animals in a volume equal to the antagonist microinjections.

Doses of the drugs were chosen based on previous studies of our research group where they have been found to be the effective doses. The use of the same doses also served the purpose of making the data comparable to the previous results.

Animals were divided into the following 4 groups, as shown in Table 1. Drugs were kept at +4 °C throughout the whole procedure.

Injection procedure. Before drug administration, mandrins serving as plugs were removed, and the delivery cannulae (27 G outer diameter, 0.4 mm) were passed through and 1 mm beyond the guide cannulae to reach the CeA. Delivery cannulae were connected to 10 µL Hamilton syringes containing the dissolved drugs via 20 cm long polyethylene tubes. The syringes were run by Cole-Parmer automatic microinfusion pump (Cole-Parmer, IITC, Life Sci. Instruments, Vernon Hills, IL, USA), which delivered the drugs evenly at a steady speed for 1 min. After the pump had stopped, the cannulae were left in place for another 1 min to allow free diffusion of chemicals and to avoid backflow of the solutions. After removing the delivery cannulae, guide cannulae were closed by mandrins again. The method allows accuracy to tenths of a millimeter administration. All drugs were microinjected bilaterally in 0.4–0.4 µL volume. Sulpiride or Veh2 pretreatment was performed 15 min before the NT or Veh1 administration. The whole procedure was performed on awake, hand-held animals.

### 2.4. Morris Water Maze Test

A circular pool 150 cm in diameter and 60 cm in height was used as the experimental apparatus of the test. The pool was filled with water stained by odorless food dye in order to avoid rats being able to see under the water level. Lamps were adjusted to provide even illumination in every part of the apparatus. Visual cues were placed around the pool to help the animals in spatial orientation. The pool was virtually divided into four quadrants. In one of them (target quadrant), a 10 × 10 cm platform was placed with its surface 1–2 cm under the water level so the rats could not see it. The platform was positioned so that the shortest distance was 50.86 cm and the longest distance was 85 cm from the wall of the apparatus. Animals were habituated to the swimming test one day prior to the experiment. Habituation meant that the rats were swimming for 90 s in the pool without the platform. Their motor activity was monitored, and those with “swimming difficulties” were excluded from further investigation. Animals were put into the pool next to the wall of the apparatus with their head towards the wall every time, and they swam to the fixed platform from this point. The start point was varied in every session, but it was the same for all animals within a trial, that is, control and treated animals needed to swim the same distance to reach the platform. The rats were staying in the pool as long as they found the platform. Those animals that could not manage this in 3 min (180 s) were placed on the platform by the experimenter. Target-finding latency (i.e., how long it took to find the safe, fixed platform) was measured with an accuracy of seconds. Furthermore, swimming velocity was measured and trajectory of movement of the animals were fixed by Noldus EthoVision program. On the first day, rats swam two times (1st and 2nd swims), and then the drugs were microinjected into the CeA. The importance of post-trial application is that this is when memory consolidation occurs [34]; furthermore, non-specific effects, such as the effect of the drug on anxiety or activity of the animals, can be excluded from the spatial learning process. On the second day, rats swam two times again (3rd and 4th swims), and the target finding latency was measured. During evaluation, the mean of 1st and 2nd and the mean of 3rd and 4th swims were used.

### 2.5. Passive Avoidance Test

The test was performed in a two-compartment passive avoidance apparatus that consisted of a well-illuminated (194 lux) bigger (60 × 60 × 60 cm) box with light grey walls and an attached smaller (15 × 15 × 15 cm), covered, dark (1 lux) box. The top of the smaller one was detachable, and a shock-grid was built on the bottom. The two boxes were separated by a trap door. The apparatus was washed and dried after each session. The passive avoidance learning test lasted for 8 days and consisted of habituation, conditioning and test sessions. Each session lasted up to 3 min. On the habituation (1st) day, animals were put into the middle of the well-illuminated box, and they were able to freely move throughout the apparatus. During conditioning, animals were placed again into the middle of the light box, and the entrance latency (i.e., how much time had passed until they entered the dark box) was measured in seconds. After the rats entered the small box, the trap door was closed, and weak electrical stimulation (0.4 mA) was performed (conditioning) three times for 1 s. Drugs were microinjected into the CeA, following which the animals were taken out from the box after the shock. Tests (tests 1 and 2) were performed 24 h and 1 week after the conditioning. Rats were put into the middle of the light box again, and the latency time to enter the dark (shock) box was measured. If the animal did not enter the dark box during the 3 min time period, the session was finished and the entrance latency was considered to be 180 s. During test 1 and test 2, animals did not receive electrical stimulus after entering the dark box.

### 2.6. Histology

Following the experiments, animals received an overdose of Calypsol and Seduxen mixed in the ratio of 4:1 and were transcardially perfused with isotonic saline followed by 10% formalin solution. After one week of postfixation, brains were frozen, cut into 40 μm serial sections and stained with cresyl violet. Injection sites were reconstructed according to the stereotaxic atlas of the rat brain [33]. Only data from rats with correctly placed cannulae were analyzed.

### 2.7. Statistical Analysis

“SPSS 13.0 for Windows” program was used for the statistical analysis of the results. Data are presented as mean ± standard error of the mean (S.E.M.). Two-way ANOVAs followed by Tukey’s post hoc analysis were employed. Statistical significance was established at *p* < 0.05. Our data were first assessed for normality using the Shapiro–Wilk test, which confirmed that our data were normally distributed.

## 3. Results

### 3.1. Histology

**Figure 1:**
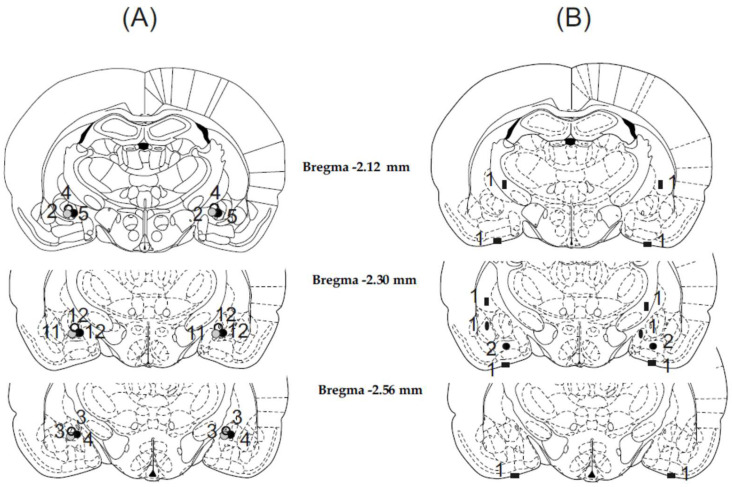
Illustration of reconstructed injection sites. Correct bilateral injection placements are indicated as closed circles in the CeA on panel (**A**) (n = 56). Incorrect injection placements are indicated on panel (**B**) (n = 8). Brain structure diagrams of coronal sections are adapted from the stereotaxic atlas of Paxinos and Watson. The numbers refer to anterior–posterior distance from bregma in mm. Identical symbols (black and grey dots and empty circles) on panel (**B**) indicate coherent injection sites of bilateral injections. Numbers above marked sites on panels (**A**) and (**B**) indicate numbers of animals.

Histological examination showed that in 56 cases of the 64 animals, the cannulae were precisely and symmetrically located on the target area (CeA). The tracks of the cannulae and tip positions were determined by evidence of debris and moderate glial proliferation. Schematic illustration of cannula placements is shown in Figure 1.

The eight rats with misplaced injection sites were excluded from subsequent analysis (Figure 1B). Among these rats, in three cases, the cannula tips were symmetrically entered into the liquor space at the basis of the brain. In two cases, cannula tips were symmetrically located 1 mm below the target area; hence, bilateral injections were made in the basomedial amygdala. In two cases, cannula tips were located laterally or medially and 1 mm above the amygdala; thus, injections were made in the caudate putamen on one side and in the internal capsule on the other side. In one case, cannula tips were placed laterally or medially to the target area; therefore, injections were made in the lateral and basolateral amygdala or in the medial amygdala nucleus. Behavioral data concerning these incorrect and diverse placements were not enough to draw far-reaching conclusions.

### 3.2. Morris Water Maze Test

**Figure 2:**
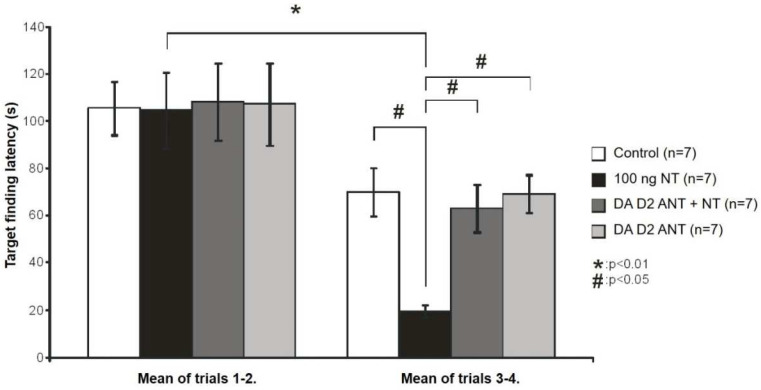
Effect of DA D2 antagonist sulpiride in Morris water maze test. Columns represent the average (+/− SEM) target-finding latencies of the first to second and third to fourth swimming trials. Control: vehicle-treated rats (n = 7); 100 ng NT: animals microinjected with 100 ng NT (n = 7); D2 ANT + NT: animals microinjected with 100 ng NT pretreated with 5 µg sulpiride (n = 7); D2 ANT: rats treated with 5 µg sulpiride (n = 7). * *p* < 0.01, # *p* < 0.05; for more explanation, see the text.

The effects of NT and DA D2 antagonist sulpiride on spatial learning were studied in the Morris water maze test (Figure 2). Two-way ANOVA analysis showed a significant difference between the sessions of the experiment [F(1, 28) = 33.839, *p* < 0.001] and among the groups receiving different treatments [F(3, 14) = 2.078 *p* < 0.05]. Moreover, the interaction between the different treatments and the sessions of the experiment was significant [F(3, 56) = 1.756, *p* < 0.05]. A post hoc test verified that finding the platform took significantly less time during the third to fourth than during the first to second swimming trial for the rats receiving 100 ng NT [100 ng NT: first to second swim vs. third to fourth swim (q = 6.881, *p* < 0.01)]. DA D2 antagonist sulpiride pretreatment inhibited the effect of NT-enhancing spatial learning, since the target finding latency of the sulpiride + NT group did not decrease significantly for the second day sulpiride + NT: first to second swim vs. third to fourth swim (q = 3.637, *p* > 0.05)]. The decrease in the target-finding latency of the sulpiride-treated group was also not significant (q = 3.481, *p* > 0.05). Swimming velocities of the animals and the distance traveled can be seen in Table 2, which show that target-finding latencies were not determined by the speed of the animals.

### 3.3. Passive Avoidance Test

**Figure 3:**
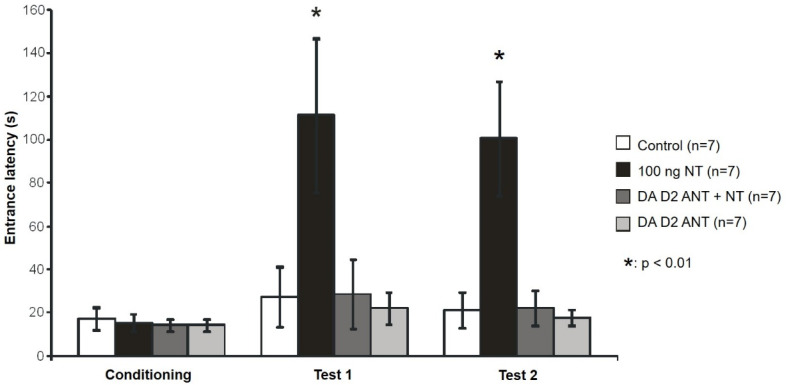
Effect of DA D2 antagonist sulpiride in passive avoidance test. Entrance latencies during conditioning, test 1 (24 h after conditioning) and test 2 trials (1 week after conditioning) are shown (+/− SEM). Control: vehicle-treated rats (n = 7); 100 ng NT: animals microinjected with 100 ng NT (n = 7); D2 ANT + NT: animals microinjected with 100 ng NT pretreated with 5 µg sulpiride (n = 7); D2 ANT: rats treated with 5 µg sulpiride (n = 7). * *p* < 0.01; for more explanation, see the text.

The effects of NT and DA D2 antagonist sulpiride on memory were examined in a passive avoidance test (Figure 3). ANOVA showed a significant difference among the sessions of the experiment [F(1, 28) = 5.011, *p* < 0.001] and among the groups receiving different treatments [F(3, 14) = 10.181 *p* < 0.001]. The interaction between the different treatments and the sessions also proved to be significant [F(3, 56) = 2.534, *p* < 0.05]. There was no inequality among the groups during the conditioning session. The entrance latency of the animals receiving 100 ng NT was significantly longer in test 1 as well as in test 2 compared to the conditioning session [100 ng NT: conditioning vs. test 1 (q = 6.38, *p* < 0.01) and 100 ng NT: conditioning vs. test 2 (q = 5.658, *p* < 0.01)]. Sulpiride pretreatment inhibited the effect of NT, since the group receiving sulpiride + NT only showed tendency to learn, and entrance latency did not increase significantly: [sulpiride + NT: conditioning vs. test 1 (q = 0.9509, *p* > 0.05)] and [sulpiride + NT: conditioning vs. test 2 (q = 0.523, *p* > 0.05)]. Animals receiving 100 ng NT achieved significantly better results during test 1 and test 2 than the other three groups [Test 1: 100 ng NT vs. control (q = 5.591, *p* < 0.01)], [Test 1: 100 ng NT vs. sulpiride + NT (q = 5.515, *p* < 0.05)], [Test 1: 100 ng NT vs. sulpiride (q = 5.943, *p* < 0.01)], [Test 2: 100 ng NT vs. control (q = 5.268, *p* < 0.05)], [Test 2: 100 ng NT vs. sulpiride + NT (q = 5.22, *p* < 0.05)] and [Test 2: 100 ng NT vs. sulpiride (q = 5.515, *p* < 0.05)].

## 4. Discussion

Several studies demonstrate that NT is involved in reinforcement and learning [12,35]. The previous findings of our research group were, however, the first to show that NT facilitates spatial learning and passive avoidance learning after being bilaterally microinjected into the rat CeA [8,10]. The involvement of NTS1 in these effects has been proven by means of NTS1 antagonist SR 48692 that was used as pretreatment and was able to block the above-mentioned actions of NT, while it had no effects in itself at the dose used [8,10]. The exact mechanisms, through which NT exerts its spatial learning and memory-enhancing effects, however, have not been revealed so far.

In the present experiments, the interaction between NT and DA in spatial learning and passive avoidance learning was tested by microinjecting 5 µg DA D2 receptor antagonist sulpiride into the CeA as a pretreatment before 100 ng NT. Since sulpiride pretreatment effectively inhibited both effects of NT, namely a significant decrease in target finding-latency in the Morris water maze test and a significant increase in entrance latency in the passive avoidance test, we conclude that the modulation of the DA system is involved in the mentioned actions of NT.

Spatial learning has been studied in the Morris water maze test, where the rats needed to find the safe platform in a pool filled with water. When animals are placed in a previously unknown environment, they form spatial memories during exploring. The hippocampus plays a critical role in this process, as well as in updating and retrieving these memories during familiarization [36,37]. It has been reported that replaying temporally sequenced information (spiking sequences) of previous behavior is an essential requirement in the mechanism of spatial navigational learning and memory [38,39,40]. Encoding experience in the new environment is related to hippocampal CA1 and CA3 place cells [41], and “replaying” was shown to occur in sleep [38,39] or during resting periods right after the spatial experience [40], that is, between swimming sessions in our experimental design when the animals received the microinjections. Although rats are known to have good swimming skills, the situation of being placed in the pool filled with water is aversive for them, so finding the safe platform where they can have a rest i a reward to them. Combining spatial locations with rewards, i.e., to maintain a representation of the location where a reward can be found, is key to survival in the natural environment of the animals. This is also proven by the fact that high-frequency oscillations called ripples (sharp-wave ripple, SWR) that occur along with the replay events usually appear around reward sites and can thus coincide with DA release [40]. There are several studies supporting that DA release may modulate memory consolidation by enhancing replay during SWR [42,43,44,45] and that inhibition of the ripples leads to spatial learning and memory impairments [46,47]. The integrity and the communication between the hippocampus and the ventral striatum (VS, including the NAC) is thought to be crucial in the development of a place–reward linkage [48]. VS receives information from several brain areas including the amygdala [49], and its neurons are facilitated by rewards [50]. It has been shown that during the replay of a learning experience, spatial aspects are activated in the hippocampus right before the emotional aspects in the VS, in order to support memory consolidation [48]. The question of how intra-amygdaloid NT can modulate the above processes is yet to be answered. It has been reported that NT facilitates the release of DA in numerous brain areas including the VTA and the nucleus accumbens [26,27,28,29,30,51], parts of the brain’s reward system. The way NT acts to result in the increased firing rate of DA neurons and DA release has not been revealed completely. NT receptors have been reported to facilitate DA neurons via a Ca^2+^-dependent mechanism [52]. Enhancing K^+^-evoked and electrically evoked DA release has also been published in the striatum [53]. A fast-scan cyclic voltammetry study suggests that NT inhibits the function of terminal D2 autoreceptors to increase DA release [27]. Our research group has recently introduced the theory that NT microinjected into the CeA may activate the DAergic neurons in the VTA that leads to the increased DA release in the limbic system, including the CeA [31,54]. We propose that information about the increased level of DA in the amygdala that is transmitted toward the VS can be reasonably supposed in the background of strengthened memory traces and enhanced spatial learning after the microinjection of NT, while sulpiride pretreatment blocks the effect of excess DA.

Regarding reinforcement learning, the role of DA has mainly been studied in reward-related tasks [55]. In the present study, the effect of NT and the involvement of DA in its action on learning and memory of the animals have been investigated in a passive avoidance test as well, where the rats were exposed to a weak electrical shock when entering the small box of the experimental apparatus. Similarly to combining spatial and reward information, learning to avoid aversive situations is also an important survival strategy of the animals, and the mesolimbic DA system is thought to be related to behaviors initiated by aversive stimuli. Behavioral responses were found to be linked to the pattern of DA signaling of the ventral medial striatum during the aversive event [56]. It has been revealed that NAC and amygdala DA transmissions are also required for active avoidance learning and maintenance of the conditioned avoidance response [57,58]. Active avoidance behavior has been shown to improve when NAC DA neurons were stimulated during the aversive cue [59]. The role of DA has been demonstrated in passive avoidance learning as well [60]. Aversive behaviors are thought to depend on neural circuits involving the thalamus, cortex, amygdala and the VS [61], and DA—based on the above—has a significant role in the regulatory process. Considering the fact that DA D2 receptor antagonist blocked the effect of NT in the passive avoidance test (significant increase in entrance latency), activation of DAergic neurons and a subsequent increase in DA level is a plausible interpretation of how NT exerts its effects in learning and memory tasks.

The results of the present investigations confirm the earlier results of our research group regarding the role of intra-amygdaloid NT in learning and memory and verify the involvement of DA D2 receptors in NT’s mechanism of action. Our results indicate that intra-amygdaloid NT-DA interaction play a role in both the reward and punishment learning paradigm.

## Figures and Tables

**Table 1:** Animal groups and treatments.

1	Control	Veh2 pretreatment + Veh1
2	NT	Veh2 pretreatment + 100 ng NT (in Veh1)
3	DA D2 ANT + NT	5 µg sulpiride (in Veh2) pretreatment + 100 ng NT (in Veh1)
4	DA D2 ANT	5 µg sulpiride (in Veh2) pretreatment + Veh1

**Table 2:** Swimming velocities and distances traveled (* *p* < 0.05).

	Mean Velocity of Trial 1–2 (cm/s)	Mean Velocity of Trial 3–4 (cm/s)	Mean Distance of Trial 1–2 (cm)	Mean Distance of Trial 3–4 (cm)
Control	26.5 ± 1.8	27.1 ± 2.1	2797.6 ± 303.8	1900.8 ± 284.3
NT	25.9 ± 1.7	26.3 ± 1.5	2711.9 ± 406.5	* 514.7 ± 70.9
DA D2 ANT + NT	27.8 ± 2.2	25.8 ± 1.9	3006.3 ± 452.8	1619.0 ± 260.6
DA D2 ANT	27.1 ± 2.5	26.5 ± 2.0	2907.3 ± 470.7	1836.4 ± 213.8

## Data Availability

Data are available at: https://drive.google.com/drive/folders/16dwVfadXB23lR28ifAgqRuhqVNHdhfwA?usp=sharing, (accessed on 1 June 2022).
